# *CAHM*, a long non-coding RNA gene hypermethylated in colorectal neoplasia

**DOI:** 10.4161/epi.29046

**Published:** 2014-05-06

**Authors:** Susanne K Pedersen, Susan M Mitchell, Lloyd D Graham, Aidan McEvoy, Melissa L Thomas, Rohan T Baker, Jason P Ross, Zheng-Zhou Xu, Thu Ho, Lawrence C LaPointe, Graeme P Young, Peter L Molloy

**Affiliations:** 1 Clinical Genomics Pty Ltd; North Ryde, NSW Australia; 2 CSIRO Preventative Health Flagship; Animal, Food & Health Sciences Division; North Ryde, NSW Australia; 3 Flinders Centre for Innovation in Cancer; Flinders University (FMC); Adelaide, SA Australia

**Keywords:** *CAHM* gene (LOC100526820), colorectal neoplasia, DNA methylation, Long non-coding RNA, Biomarker, circulating DNA, *QKI*

## Abstract

The *CAHM* gene (*Colorectal Adenocarcinoma HyperMethylated*), previously LOC100526820, is located on chromosome 6, hg19 chr6:163 834 097–163 834 982. It lacks introns, encodes a long non-coding RNA (lncRNA) and is located adjacent to the gene *QKI*, which encodes an RNA binding protein. Deep bisulphite sequencing of ten colorectal cancer (CRC) and matched normal tissues demonstrated frequent hypermethylation within the *CAHM* gene in cancer. A quantitative methylation-specific PCR (qMSP) was used to characterize additional tissue samples. With a threshold of 5% methylation, the *CAHM* assay was positive in 2/26 normal colorectal tissues (8%), 17/21 adenomas (81%), and 56/79 CRC samples (71%). A reverse transcriptase-qPCR assay showed that CAHM RNA levels correlated negatively with *CAHM* % methylation, and therefore *CAHM* gene expression is typically decreased in CRC. The *CAHM* qMSP assay was applied to DNA isolated from plasma specimens from 220 colonoscopy-examined patients. Using a threshold of 3 pg methylated genomic DNA per mL plasma, methylated *CAHM* sequences were detected in the plasma DNA of 40/73 (55%) of CRC patients compared with 3/73 (4%) from subjects with adenomas and 5/74 (7%) from subjects without neoplasia. Both the frequency of detection and the amount of methylated *CAHM* DNA released into plasma increased with increasing cancer stage. Methylated *CAHM* DNA shows promise as a plasma biomarker for use in screening for CRC.

## Introduction

There is an unmet need for new diagnostic biomarkers that can improve the detection of adenomas and colorectal cancer (CRC) in a cost-effective manner for population screening programs. Genes whose expression change markedly in association with colorectal neoplasia are therefore potential biomarkers, especially if the change can be detected in a non-invasive patient sample such as blood or stool. Changes in gene expression may be accompanied by changes in DNA methylation at the gene locus and abnormal methylation of genomic regions, such as promoters, CpG islands and CpG island “shores,” is a common feature of many cancer types, including CRC,^1^^,^^2^ providing the opportunity for development of assays for cancer detection.^3^^,^^4^ In CRC, the methylation levels of specific genes may serve as cancer biomarkers; for example, methylation-specific assays have been developed to detect cancer-derived *SEPT9* and *VIM1* DNA in blood and fecal samples, respectively.^5^^,^^6^ The advent of methods for genome-wide analysis of DNA methylation has provided an opportunity to survey the genome comprehensively for cancer-associated alterations that occur with high frequency. This has stimulated a search for new diagnostic biomarkers, as well as for individual genes or multi-gene panels whose DNA methylation status might allow tumor classification, prognosis, and the prediction of likely response to different treatment options. Moreover, since the identification and removal of colorectal adenomas prevents their progression to CRC,^7^^,^^8^ the early detection of colorectal neoplasia in population screening could actually lower the incidence of CRC.^9^

In preliminary analysis of data collected using a new genome-wide method to inspect DNA methylation levels, we identified cancer-specific hypermethylation at a TaqI site within a previously uncharacterized human RefSeq gene, LOC100526820, on the minus strand at chr6:163 834 097–163 834 982 (hg19 coordinates). As seen in Figure S1, this gene is located adjacent to the *QKI* gene, which is on the plus strand and which was included in a set of genes that we had previously identified as being downregulated in CRC and adenomas (Supplementary Table 1 of ref. 10). *QKI* was also recently proposed to be a tumor suppressor gene whose expression is decreased in CRC, a change accomplished at least partially by DNA methylation.^11^ Our interest in this uncharacterized gene was further heightened when it was classified as a long non-coding RNA, particularly in the context that the neighboring *QKI* gene encodes an RNA binding protein. lncRNAs are increasingly implicated in cancer^12^ and are potentially useful for cancer diagnosis.^13^ Examples of lncRNAs that are upregulated in colorectal neoplasia have recently come to light, e.g., *CRNDE*^14^ and HOTAIR.^15^

LOC100526820 has now been given the gene symbol *CAHM*, for *Colorectal Adenocarcinoma HyperMethylated*. On bioinformatics grounds (including the lack of a viable open reading frame) the gene is considered not to encode a protein, is classified as “ncRNA” in the NCBI Gene database (http://www.ncbi.nlm.nih.gov/gene/?term=CAHM%20gene) and is assigned a lncRNA identifier (LINC00468). The RefSeq gene locus comprises a single exon and CAHM transcripts are unspliced; this, along with ENCODE data describing the histone marks and transcription factor binding at the locus are shown in Figure S1.

In this paper, we first describe an examination of the methylation level across the LOC100526820/CAHM gene locus in normal and neoplastic colon tissue specimens. Having developed a methylation-specific PCR (qMSP), we then demonstrate that *CAHM* is methylated at the sites of the qMSP primers in a high proportion of colorectal adenomas and cancers, but not in normal colorectal tissue. We observe that the hypermethylation of *CAHM* in colorectal adenomas and CRC is accompanied by a decrease in its transcription; this downregulation contrasts with elevated transcription of lncRNAs such as CRNDE and HOTAIR in CRC. We further demonstrate that methylated *CAHM* sequences can be detected in the plasma of patients with CRC, and thus may potentially contribute to assays for the detection of CRC using non-invasive samples.

## Results

### Identification of *CAHM,* a novel gene hypermethylated in colorectal adenocarcinoma

“Bisulphite-tagging,” a method that interrogates the methylation status of the CpG site within TaqI (5′-T′CGA) and MspI (5′-C′CGG) restriction sites in the genome, was applied to DNA isolated from three CRC cell lines, SW480, HCT116, and LIM1215, as well as to DNA from eight pairs of CRC and matched normal tissue samples^10^^,^^16^. Among the CpG sites methylated in CRC tissue and cell lines, but not in normal tissue, was a cytosine lying within the previously uncharacterised RefSeq gene LOC100526820. The cytosine, part of a TaqI restriction site, had the coordinate chr6:163 834 406 (Fig. 1A). LOC100526820 is located adjacent to the *QKI* gene, but transcribed in the opposite direction (Fig. 1).

**Figure:**
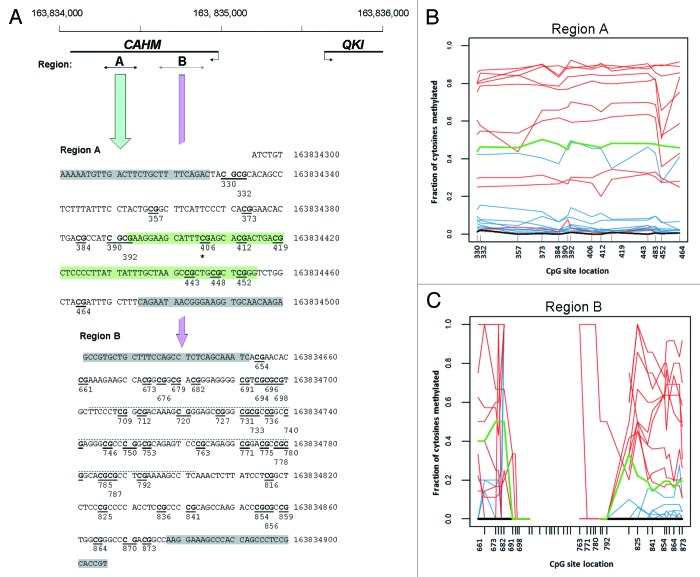
**Figure 1.** The *CAHM* gene locus (LOC100526820).****(**A**) The region on chromosome 6 encompassing the *CAHM* gene and the first exon of the adjacent *QKI* gene. Arrows show the directions of transcription. Double-headed arrows show the positions of two regions selected for bisulphite sequencing: Region A spanned chr6:163,834,295–163,834,500 (left double-headed arrow, black), Region B spanned chr6:163 834 621–163 834 906 (center double-headed arrow, gray). The nucleotide sequences of Regions A and B are those on the plus strand of the chromosome. Primer locations are highlighted in gray, and interrogated CpG sites (bold, underlined) are numbered with the last three digits of their chromosomal nucleotide position. The differentially methylated cytosine in the TaqI site from the initial Bisulphite-tag survey is marked by an asterisk (*). The segment used later as template for the methylation-specific qPCR assay is highlighted in green, while a dotted line has been placed above that used later in RT-qPCR for CAHM expression The graphs (right) show the fraction of methylated cytosines at (**B**) the 15 individual CpG sites in Region A and (**C**) the readable sites in Region B (identified by the last three digits of their chromosomal nucleotide position) for each of the 10 matched CRC (red) and normal (blue) samples. Also shown is the result for the WBC DNA (black) and a 50:50 control mix (green).

Bisulphite conversion-specific deep sequencing was used to determine the level of methylation in two chromosomal regions: chr6:163 834 295–163 834 500 (“Region A”) and chr6:163 834 621–163 834 906 (“Region B”), the former of which overlaps the originally identified TaqI site (Fig. 1A). Sequencing was done on bisulphite-converted DNA extracted from ten previously untested CRC and matched normal tissue specimens.

For Region A, between 100 and 600 reads of individual bisulphite-treated strands were obtained for each sample. The sequencing revealed that 7 of the 10 CRC samples showed high levels of methylation (60–90%) at the majority of CpG sites (Fig. 1B); of the remaining three samples, two showed intermediate levels (30–50%) and one showed minimal (<10%) methylation. In contrast, 8 of the 10 normal DNA samples showed methylation of less than 10% at CpG sites across Region A (Fig. 1C); of the remaining two, one displayed low levels (10–20%), and the other, intermediate levels (~40%). The CRC tissue sample from the patient with ~40% methylation in matching normal tissue was one of those showing very high methylation (80–90%), indicating that a further increase had still occurred in neoplasia. The sequencing also revealed that commercially-sourced WBC DNA (Roche) contained less than 3% methylation at all CpG sites in Region A (Fig. 1B), and could therefore be used as an unmethylated DNA control.

The sequencing data obtained from Region B was of poor quality, probably due to its high GC content (73%) and data points are only shown where there were >10 reads at a CpG site. Despite this, high methylation of the ten CpGs in the final 70 bp of the amplicon (i.e., closest to the presumed TSS of *CAHM*) was clearly seen in CRC but not in normal samples (Fig. 1), indicating that CRC-associated methylation is not unique to Region A but most likely extends across the whole *CAHM* gene locus. Segments upstream of Region A were also investigated but failed to yield any sequence data, probably because of their very high GC content (e.g., the 200 nt surrounding the presumed *CAHM* TSS at the 5′ terminus of the locus is 81% GC, while the corresponding segment at the *QKI* TSS is 83% GC).

Based on the relationship between high *CAHM* methylation and CRC revealed by the bisulphite-tag and bisulphite deep sequencing data, the HUGO Gene Nomenclature Committee (HGNC) approved renaming of LOC105526820 as *Colon Adenocarcinoma HyperMethylated* (non-protein coding), gene symbol *CAHM*.

### *CAHM* hypermethylation in neoplastic colorectal tissue

A quantitative methylation-specific qPCR assay (qMSP) for *CAHM* was developed whose primers span six CpG sites in the *CAHM* locus (Fig. 2A). After optimization of the assay, a plot of Ct values vs. amount of methylated DNA demonstrated good linearity (R^2^ = 0.9584) down to 20 pg and sensitivity down to 5 pg of methylated DNA (equivalent of 1–2 genomic copies) in a total of 5 ng DNA (Fig. 2B). The assay only rarely gave a signal for WBC DNA, which is essentially unmethylated (Fig. 2B).

**Figure:**
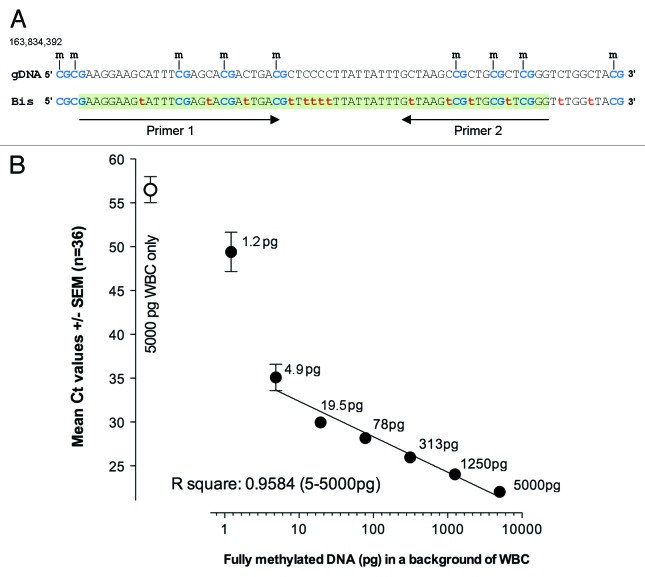
**Figure 2.** Methylation-specific qPCR assay for *CAHM*. (**A**) Top strand: fully methylated DNA, with CpG sites shown in blue capitals; m = methyl group. Bottom strand: bisulphite-converted equivalent, with thymidines derived from unmethylated cytosine nucleotides shown in red lower-case. Arrows: positions and sequences of the methylation-specific oligonucleotide primer pair (Table S2, PCR assay ID 3). (**B**) Standard curve (filled circles) for the *CAHM* methylation-specific qPCR assay, shown as a plot of mean Ct values ± SEM (n = 36 for each point) for a 4-fold serial dilution of positive control methylated DNA in negative control unmethylated DNA. Open circle: 31 of the 36 WBC DNA samples gave no signal in the *CAHM* qPCR assays and an arbitrary value of “60” was assigned to these negative samples. Thus, the data point is the mean value of 5 measured Ct values and 31 negative “60” values.

The extent to which *CAHM* was methylated at the qPCR target sites was measured in bisulphite-converted DNA extracted from 126 colorectal tissue specimens comprising 26 normal, 21 adenoma and 79 CRC, the cancer samples spanning Stages I to IV. A qMSP result indicating that 5% or more of the DNA in the sample was methylated at the sites targeted by the PCR primers was considered to be positive, and this was taken to indicate that *CAHM* was substantially methylated (i.e., hypermethylated) in that sample. The proportion of methylated *CAHM* (% methylation) was substantially higher in neoplastic samples (i.e., adenoma and CRC) than in normal colorectal tissue. With the threshold of 5% methylation, the *CAHM* assay was positive in 2/26 normals (8%), 17/21 adenomas (81%), and 56/79 cancers (71%) (Table 1). The proportion of methylated *CAHM* was statistically significantly higher in adenomas and CRC Stages II-IV than in normal colorectal specimens (*P* < 0.0002), with median *CAHM* methylation levels of 17–40% in neoplastic colorectal specimens (Fig. 3). Hypermethylation of CAHM was observed in a high fraction (17/21) of adenoma samples, indicating that this is a common early event in CRC development.

**Table:** **Table 1.** Prevalence of methylated *CAHM* in DNA from tissue and plasma samples

		Tissue^a^				Plasma^a^		
		*CAHM* ≥ 5% methylation				meCAHMeqgDNA ≥ 3 pg/ml		
Tissue	Disease stage	Total (n)	Matched (n)	Positive (n)	Positive %	Total (n)	Positive (n)	Positive %
Colorectal	Normal	26	6	2	8	74	5	7
	Adenoma	21	1	17	81	73	3	4
	CRC Stage I	20	4	10	50	12	5	42
	CRC Stage II	21	1	15	71	21	11	52
	CRC Stage III	30	0	25	83	23	12	52
	CRC Stage IV	8	0	6	75	12	9	75
	CRC unknown	-	-	-	-	5	3	60
Lung	Normal	10	10	0	0	-	-	-
	Cancer	10	10	0	0	-	-	-
Prostate	Normal	5	5	0	0	-	-	-
	Cancer	5	5	0	0	-	-	-
Breast	Normal	10	10	0	0	-	-	-
	Cancer	10	10	2	20	-	-	-
Colon	Normal	10	10	1	10	-	-	-
	Cancer	10	10	8	80	-	-	-

^a^ Tissue and plasma samples are from different cohorts of patients; - indicates not applicable or no data available.

**Figure:**
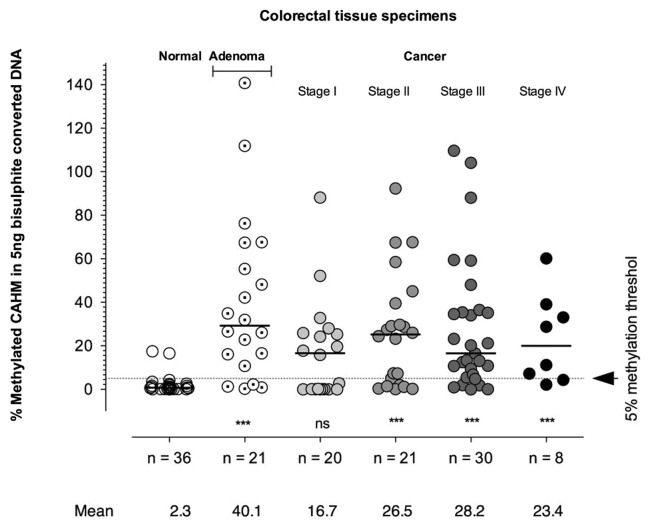
**Figure 3.** Prevalence of methylated *CAHM* DNA in normal and neoplastic colorectal tissue. The *CAHM* qMSP assay (Table S2, PCR assay ID 4) was used to measure the proportion of methylated *CAHM* in 5 ng bisulphite-converted DNA from 126 colorectal tissue specimens including normal (open circles, n = 26), adenoma (dotted open circles, n = 21) and 79 CRC (light gray: Stage I, n = 20; medium gray: Stage II, n = 21; dark gray: Stage III, n = 30; black: Stage IV, n = 8), horizontal bars = median. Dotted line represents 5% methylation, and the count of specimens with ≥5% *CAHM* methylation is summarized in Table 1. The mean levels of methylation for each of the 6 tissue specimen groups are given at the bottom of the figure. ***P value < 0.0002; ns, non-significant, relative to normal.

For DNA from 11 adenomas and 68 cancer samples, we also measured the proportion of the *SEPT9* gene that was methylated (Table S3). While *SEPT9* was more frequently methylated in CRC, *CAHM* was methylated in 8 of 11 adenomas compared with 7 of 11 for SEPT9 (5% threshold for positives), and for 3 of the 11 adenomas the proportion of *CAHM* that was methylated was at least 2-fold higher than the corresponding value for *SEPT9*. This was also the case for one Stage I and one Stage II cancer. The substantially higher level of methylated *CAHM* in some neoplastic samples, particularly adenomas and early stage cancers, suggests that its diagnostic use in combination with *SEPT9* may add to the sensitivity of detection of colorectal neoplasia-derived DNA.

### *CAHM* methylation correlates with of loss expression of CAHM RNA in colorectal tissue and CRC cell lines

To investigate transcription from the *CAHM* locus, we used a primer pair targeting a region (dotted line in Fig. 1A) in the less GC-rich 3′- half of the predicted CAHM RNA transcripts. RT-qPCR confirmed the presence of CAHM transcripts in RNA extracted from normal colorectal tissue (Fig. 4A).

**Figure:**
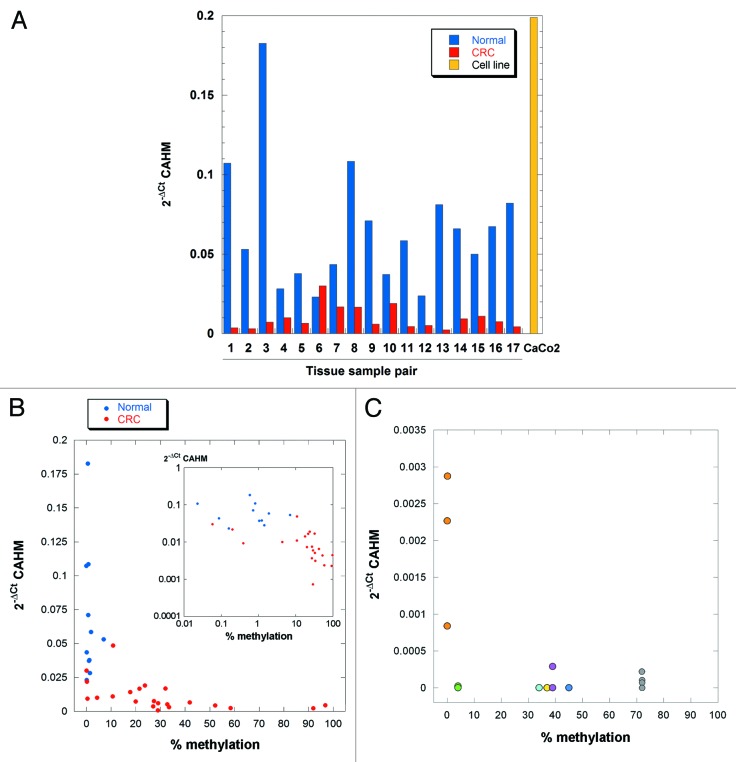
**Figure 4.** Expression of CAHM RNA in colorectal tissue. (**A**) Histogram showing CAHM transcript expression in colorectal tissue samples (normal, blue; CRC, red), as measured by RT-qPCR (relative to HPRT1 expression). For comparison, the level observed in the highest-expressing CRC cell line (CaCo2, orange) is also shown. (**B**) CAHM expression in colorectal tissue samples (normal, blue; CRC, red) plotted against the corresponding proportion of methylated *CAHM*, as measured by the qMSP assay (relative to HPRT1 expression). The inset graph shows the data replotted on a log-log scale. (**C**) CAHM expression in CRC cell lines plotted against *CAHM* methylation, as measured by qMSP (relative to β-actin expression). Fill colors are CaCo2, orange (as in panel **A**); LS174T, green; HT29, cyan; LIM1215, orange; SW480, magenta; SW620, blue; HCT116, gray.

In all but one tissue pair (n = 17), we observed a decrease in *CAHM* transcription in CRC relative to matched normal colorectal tissue (Fig. 4A); the exception had similar levels in both samples. In Figure 4A, the median relative expression values for CAHM changed from 0.0586 (normal) to 0.0073 (CRC), representing an 8.0-fold and significant difference (*P* < 0.0001 in paired *t* test; n = 34). The expression of CAHM RNA in colorectal tissue was seen to correlate negatively with the extent of *CAHM* gene methylation, with a highly nonlinear dose-response relationship (Fig. 4B). In CRC cell lines, CAHM expression again correlated negatively with *CAHM* methylation in a nonlinear fashion (Fig. 4C). Specifically, CaCo2—the only cell line with no *CAHM* methylation—showed significant CAHM expression, comparable to the highest levels seen in normal colorectal tissue (Fig. 4A), whereas all of the other cell lines (for which ≥3% of the qMSP target regions were methylated) showed little or no expression (Fig. 4C). The highest observed levels of CAHM RNA expression, namely those in normal colorectal tissue and in CaCo2 cells, were still less than 30% of HPRT or <0.3% of β-actin mRNA expression (Fig. 4A and C).

### *CAHM* hypermethylation in other cancer types

To evaluate whether hypermethylated *CAHM* was specific for colorectal neoplasia, the methylation status of *CAHM* was measured in matched cancer/normal tissue specimens from lung (10 pairs), prostate (5 pairs), and breast (10 pairs), as well as 10 pairs of previously untested CRC/normal specimens. With the same threshold as used above (5% of qMSP targets methylated), *CAHM* was validated as hypermethylated in 8/10 (80%) and 1/10 (10%) matched CRC and normal specimens, respectively (Table 1). *CAHM* was found to be hypermethylated in only 2 other cancer specimens, namely breast cancer (2/10, 20%), whereas it was not methylated in lung or prostate cancer samples. Only 1 of the 35 normal samples in the panel—a colorectal tissue specimen—tested positive (3%).

### Increased levels of hypermethylated *CAHM* in blood from CRC patients compared with normals

The high proportion of methylated *CAHM* DNA seen in most CRC specimens and the low proportion of methylated *CAHM* seen in most healthy tissue specimens and in WBC DNA led us to investigate whether the level of methylated *CAHM* DNA in non-invasive samples (such as blood fractions) could be used to distinguish CRC patients from healthy individuals. The potential for background levels of methylated DNA (e.g., from white blood cells) to cause false positives in blood from healthy subjects is a major concern in such assays^17^. We therefore explored the detection of methylated *CAHM* sequences in bisulphite-converted DNA extracted from plasma of 220 patients who were classified by colonoscopy to include 74 normal patients, 73 with adenoma and 73 with CRC. Each of the triplicate assays contained DNA isolated from the equivalent of 0.55 mL of plasma. The level of methylated *CAHM* sequences measured in each plasma sample is shown in Figure 5. Using a threshold of 3pg meCAHMeqgDNA per mL plasma, we obtained a positive result with 5/74 (7%) normal patients, 3/73 (4%) patients with colorectal adenomas, and 40/73 (55%) CRC patients (Table 1). Sixty-nine of 74 plasma specimens collected from healthy controls yielded no detectable methylated *CAHM* (Table 1), corresponding to a specificity of 93%.

**Figure:**
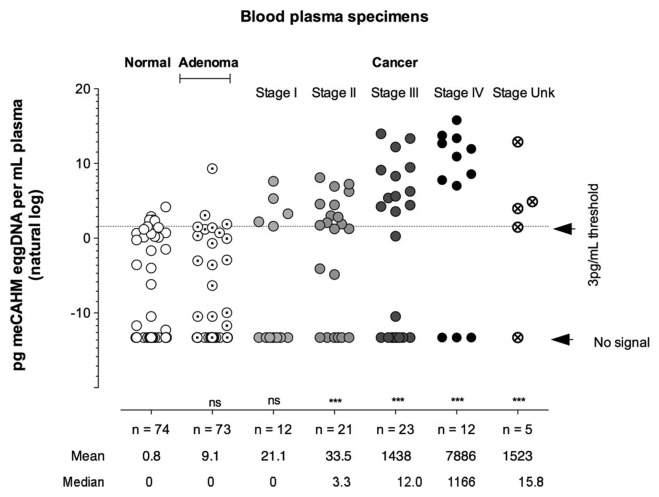
**Figure 5.Level of hypermethylated *CAHM* DNA in blood plasma.** The *CAHM* qMSP assay (Table S2, PCR assay ID 4) was used to measure of the level of *CAHM* methylation in bisulphite-converted DNA extracted from the equivalent of 0.55 mL plasma (triplicates) from 220 plasma samples obtained from colonoscopy-confirmed patients, of whom there were 74 normal (open circles), 73 adenoma (dotted circles) and 73 CRC (light gray: Stage I, n = 12; medium gray: Stage II, n = 21; dark gray: Stage III, n = 23; dark: Stage IV; crossed circles: unknown Stage, n = 5). Data points are mean pg meCAHMeqgDNA per mL plasma. Dotted line: 3 pg/mL threshold. The number of specimens with >3 pg/mL meCAHMeqgDNA is summarized in Table 1. The mean levels of methylation for each of the seven phenotypic groups of patients are given at the bottom of the figure. ***P value < 0.0002; ns, non-significant, as compared with normal.

### Model for estimation of class probabilities—*CAHM* methylation levels in blood reflect disease severity

A statistically significant increase in the estimated mass of methylated *CAHM* DNA in plasma drawn from patients with Stage II, III to IV CRC was observed, relative to normal plasma (Fig. 5). We sought to model the relationship between methylated *CAHM* levels in plasma and cancer stage, and to estimate the likelihood of a particular cancer stage for a given meCAHMeqgDNA value. The corresponding distribution profiles were calculated (Fig. 6A) using meCAHMeqgDNA values for plasma specimens from patients with known phenotypes (as shown in Fig. 5, but omitting the “no signal” results), including the phenotype classes: normal, adenoma, early stage CRC (= Stage I + Stage II) and late stage CRC (= Stage III + Stage IV). The modeled density plots, assuming the observed methylation levels were drawn from a normal distribution (Fig. 6B), were used to estimate the cumulative probability that a plasma sample of a known classification would yield an observed meCAHMeqgDNA amount such that its natural log exceeds 1.0, 2.0, and 5.0 (Fig. 6C and dotted lines and top arrows in Fig. 6B). We determined that only 3% of premalignant (= normal + adenoma) plasma specimens are found to contain 148 pg or more of meCAHMeqgDNA, whereas 66% of late stage CRC show 148 pg or more. Thus the likelihood of a plasma specimen with ≥148 pg meCAHMeqgDNA to be a late stage CRC is 23:1 (late stage: premalignant). Further, a plasma specimen with 22 ng of meCAHMeqgDNA is approximately 1600 times more likely to be drawn from a patient with late stage CRC than from a healthy patient or one with premalignant neoplasia.

**Figure:**
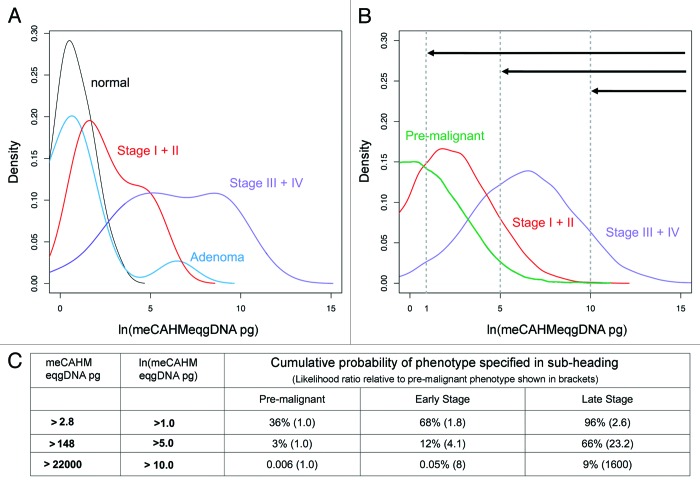
**Figure 6.** Estimation of class probabilities. (**A**) Empirical probability density plots (omitting “no-signal” results) from the 215 tested plasma samples (Fig. 5) with known phenotypes including colonoscopy-confirmed normal (black, 5 CAHM positives), adenoma (blue, 3 *CAHM* positives), early stage CRC (red, Stage 1+2, 16 *CAHM* positives), late stage CRC (purple, Stage 3+ 4, 21 *CAHM* positives). (**B**) Modeled density plots assuming a normal distribution of phenotypic classification including pre-malignant (green, normal + adenoma), early stage CRC (red, Stage 1+2) and late stage CRC (purple, Stage 3+4). (**C**) Cumulative probability of a plasma specimen with *CAHM* methylation levels equal to or greater than 2.8, 148, or 22,000 pg meCAHMeqgDNA coming from a patient with the indicated phenotypic classification; the mass thresholds were determined as the area under the curve (Panel **B**) for ln(pg meCAHMeqgDNA) values greater than 1, 5, and 10, respectively (see dotted lines and top arrows in Panel **B**). Likelihood ratios relative to pre-malignant are shown in brackets.

## Discussion

The gene name *Colon Adenocarcinoma HyperMethylated (non-protein coding),* gene symbol *CAHM,* has been assigned to LOC100526820 (hg19 chr6:163 834 097–163 834 982) based on the results described above, which demonstrate increased methylation in colorectal neoplasia relative to normal colon mucosa. The *CAHM gene* encodes a long non-coding RNA (lncRNA), RefSeq NR_037593, and is located about 730 bp upstream of the adjacent gene, *QKI*, which is on the opposite strand. A recent ENCODE survey revealed that the expression of almost 3% of lncRNAs shows high positive correlation with that of a neighboring mRNA,^18^ and we note that *QKI* is also downregulated in colorectal neoplasia^11^^,^^19^ and that this is associated with hypermethylation.^11^ Yang et al.^11^ and Novikov et al.^20^ have provided evidence that *QKI* acts as a tumor suppressor and that its downregulation may be significant in cancers from several different organs. The genomic region encompassing both *CAHM* and *QKI* is reported to be commonly deleted in glioblastomas.^21^

The gene product Qki has been shown to be an RNA-binding protein^22^^,^^23^ and a deficiency of Qki protein is associated with developmental defects in neural and vascular tissues.^24^^,^^25^ There is evidence for its tumor-suppressive actions being mediated through two pathways: (1) regulation of alternate splicing, e.g., regulating the levels of alternate splice forms of the histone Macro-H2A,^20^ and (2) through stabilization of the microRNA mir20-a, which regulates genes in the TGF-β pathway.^26^ The lncRNA Gomafu/MIAT has recently been shown to bind to Qki and modulate its splicing specificity.^27^ Given the close proximity of *CAHM* to *QKI*, we speculate that CAHM transcripts might also be RNA binding partners for the Qki protein and may perhaps be involved in directing its specificity. Silencing of *CAHM,* in addition to *QKI*, may therefore be important for tumor development.

In tissues from CRC patients, we have demonstrated through deep bisulphite sequencing that, for almost all patients, there was little or no methylation of CpG dinucleotides within the *CAHM* gene in DNA from histologically normal mucosa, whereas the locus was hypermethylated in a high proportion of colorectal neoplasias. This methylation is present in the body of the *CAHM* gene and extends (at least) from just after the presumed TSS to the center of the 3′ half of the gene. When our data are combined with those of Yang et al.,^11^ methylation would appear to extend across the CpG island and encompass both the *CAHM* and *QKI* TSS. Based on our methylation data, we developed a methylation-specific PCR assay (qMSP) that has a detection limit of approximately 5 pg meCAHMeqgDNA, an amount corresponding to the DNA content of a single diploid cell (6.6 pg). Its sensitivity is therefore similar to that of commercial assays, such as that for methylated *SEPT9* sequences.^6^ In addition to observing frequent (56/79, 71%) hypermethylation of *CAHM* in DNA from CRC tissues relative to that from normal controls (Table 1), we report that most (17/21, 81%) DNA samples from colorectal adenoma tissues likewise show hypermethylation of *CAHM*.

Using the qMSP assay and an RT-qPCR assay to measure *CAHM* methylation and transcript abundance, respectively, we demonstrate that hypermethylation of *CAHM* in CRC correlates with a decrease in CAHM transcription (Fig. 4A and B). The dose-response relationship is more switch-like than linear, with little or no expression (in either CRC cell lines or colorectal tissue) occurring above 15% methylation (Fig. 4B and C). While methylation within the gene body has often been seen to correlate with elevated gene expression^28^^,^^29^^,^ this is generally not associated with regions of high CpG density. In our case, it seems likely that high % methylation of Regions A and B extends across the whole CpG island including at the *CAHM* and *QKI* promoter regions, with hypermethylation leading to the epigenetic silencing of both genes. The very high GC content of this upstream region has prevented us from assaying its methylation status directly.

Examination of a broader tissue set including lung, prostate and breast cancers demonstrated significant specificity for methylation of *CAHM* in CRC relative to other cancers (Table 1). The frequent hypermethylation of *CAHM* in adenomas suggests that this biomarker is hypermethylated relatively early in colorectal oncogenesis. The fact that *CAHM* is sometimes much more heavily methylated than *SEPT9* in colorectal neoplasia (Table S3) suggests that *CAHM* methylation may be usefully combined with that of *SEPT9* for detecting disease at the earliest stages.

Limitations of the study include the fact that methylation was measured in DNA extracted from a heterogeneous population of cells in resected tissue. Moreover, although the initial deep bisulphite sequencing exercise and the subsequent validation of the qMSP assay used matched CRC/normal pairs (10 pairs each), the colorectal tissue samples on which the bulk of work was conducted (21 adenoma and 89 CRC) included only 16 paired samples (i.e., where normal and neoplastic samples came from the same subject, Table 1). While we have investigated the correlation between *CAHM* methylation and disease stage, we have not looked for correlations with cancer subtypes (e.g., deficienct *MLH1* DNA mismatch repair and microsatellite instability; CIMP status and associated BRAF mutations; and the presence of other classic PI3K/MAPK or Wnt pathway mutations).

When applied to DNA isolated from plasma, with a threshold of 3 pg meCAHMeqgDNA/mL plasma (Table 1), we found positive *CAHM* methylation in 40 individuals of the 73 CRC patients tested (55%) and only 5 of 74 controls (7% false positives). The mean level of methylated *CAHM* DNA in plasma of CRC patients was 1893 pg meCAHMeqgDNA/mL (175–3611 pg/mL, 95% CI). These results suggest that CRC patients carry a mean burden of ~300 cell equivalents of cancer-derived DNA/mL plasma (Table S1). The high specificity of the assay (93%) could be a particularly attractive feature in CRC screening, as it would have the potential to reduce the number of unnecessary colonoscopy follow-up procedures.

Interestingly, the frequent hypermethylation of *CAHM* observed in colorectal adenoma tissues is not reflected in the plasma of adenoma patients. We hypothesize that the discrepancy between adenoma tissue-plasma correlation and CRC tissue-plasma correlation may be related to the higher architectural distortion and vascularity of cancer tissues, as well as greater lesion size, compared with adenomas. With a higher perfusion level for cancers compared with adenomas, DNA from cancers may more readily enter the circulation and thus be more readily detectable in plasma. Supporting this hypothesis, an analysis of the amount of methylated *CAHM* in DNA extracted from plasma relative to CRC staging showed that increasing levels of methylated *CAHM* correlated with advancing disease stage in these limited data (Fig. 5). Future studies could investigate the proportion of methylated *CAHM* as a function of the different subtypes of CRC and the clinical or phenotypic characteristics of the individuals in whom these cancers arise.

By fitting models of cancer classification (no CRC, early or late CRC), we show the potential of using the level of methylated *CAHM* sequences in blood to estimate the likelihood of a positive result being due to the presence of CRC (Fig. 6). It is not yet clear whether the release of pre-malignant cells (or their DNA) from adenomas might be more readily detected in fecal samples. With the potential for further assay improvements, particularly in the context of using panels of biomarkers to improve sensitivity, *CAHM* hypermethylation provides a biomarker worthy of future study and with promise to enhance the early detection and inform treatment of CRC.

## Materials and Methods

### Specimen collection

All tissue and blood specimens were obtained from consenting individuals. Ninety-six colorectal tissue specimens obtained from surgical resections were fresh-frozen and stored at –80 °C at a tertiary referral hospital tissue bank in Adelaide, South Australia. Access to the tissue bank for this research was approved by the Research and Ethics Committee of the Repatriation General Hospital (Adelaide) and the Ethics Committee of Flinders Medical Centre. Colorectal tissue specimens were classified as normal (n = 26), adenoma (n = 21) or adenocarcinoma (n = 87) on the basis of histological assessment by an expert pathologist. An additional panel of matched cancer/normal tissue sample pairs from a range of tissue types (10 pairs for each of lung, breast and colon; 5 pairs for prostate) was purchased from Proteogenex.

Blood plasma specimens were acquired from a commercial specimen bank (Proteogenex), who generated them as follows: Peripheral blood was drawn into K_3_EDTA VACUETTE blood tubes (Greiner-One, Preanalytics Cat. 456036) and transported to the processing laboratory on wet ice. Whole blood was centrifuged at 1500 g (4 °C) for 10 min within 4 h of blood draw and plasma was recovered. The plasma was centrifuged for a second time at 1500 g (4 °C) for 10 min, whereafter the supernatant (plasma) was collected and stored at -80 °C until further use. Blood specimens were classified as normal (n = 74), adenoma (n = 73) or CRC (n = 73) based on colonoscopy results and verified by histopathology in the case of adenoma or cancer. Phenotypic characteristics of all patients are listed in Table S1.

### Tissue DNA extraction and bisulphite conversion

Fresh-frozen tissue specimens were homogenized using a bead homogenizer and genomic DNA extracted using a Wizard^®^ Genomic DNA Purification Kit (Promega, Cat. A1120). An additional set of DNA extracted from 30 colorectal tissue specimens (10 normal, 10 adenoma, and 10 CRC) was commercially sourced through Bioserve Biotechnologies. DNA from cell lines was also extracted using Wizard^®^ Genomic DNA Purification Kit. The DNA concentration was determined by Nanodrop ND 1000 spectrophotometer (Nanodrop Technologies) and 1 μg of DNA was bisulphite converted using either the EZ DNA Methylation-Gold Kit (Zymo Research Corp., Cat. D5005) or EpiTect Fast 96-well Bisulphite Conversion Kit (QIAGEN, Cat. 59720) as recommended by manufacturers, but with modified bisulphite reaction temperature conditions for the Zymo kit.^10^ The concentration of bisulphite-converted DNA was determined by quantitative real-time PCR using bisulphite conversion-specific primers for either *ACTB* or CFF1^5^ as described in Table S2, PCR assay IDs 4 and 5, respectively. The bisulphite-converted tissue DNA samples were stored at –80 °C until further use. Enzymatically methylated human DNA (CpGenome Universal Methylated DNA, Millipore, Cat. S7821) was used as a positive methylation control, and white blood cell DNA (WBC DNA, Human Genomic DNA from human buffy coat, Roche Applied Science, Cat. 1169111201) as an unmethylated control.

### Plasma DNA extraction and bisulphite conversion

DNA was extracted from 4 mL plasma using the “QIAamp Circulating Nucleic Acid Kit” (QIAGEN, Cat. 55114). The manufacturer’s protocol was followed except that the column was washed twice with ACW2 and twice with 100% ethanol, after which DNA was eluted by applying 35 μL of AVE buffer twice over the column. This optimized protocol resulted in a higher DNA yield (data not shown). A total of ~33 μL DNA was obtained per 4 mL plasma extraction and approximately 31 μL was incubated at 37 °C for 1 h in a final reaction volume of 35 μL containing 2µg/mL tRNA (*E. coli*), 280 µg/mL Proteinase K and 1% SDS. The samples were subsequently bisulphite-converted using the EZ DNA Methylation-Gold Kit as recommended by the manufacturer (Zymo) with the same modification to cycling conditions described above. The bisulphite-converted DNA was eluted using 40 μL nuclease-free water, resulting in a final volume of 36 μL. Duplicate 2μL aliquots were analyzed in the ACTB qPCR assay (as above) to calculate concentration of recovered bisulphite-converted DNA. DNA recovered ranged from 1.1 to 68 ng/mL plasma (mean 9.5 ng, median 5.6 ng/mL plasma). Triplicate 5 μL aliquots of bisulphite-converted DNA extracted from 4mL plasma (the equivalent of 555 μL plasma in each) were analyzed using the *CAHM* methylation-specific qPCR assay as described below and in Table 1.

### Sequence analysis of bisulphite-treated DNAs

All nucleotide numbering in this paper is specified in hg19 coordinates. Two regions were amplified from bisulphite-treated DNA: Region A spanned chr6:163 834 295–163 834 500 and Region B spanned chr6:163 834 621–163 834 907 (Fig. 1). Amplification of 10 ng bisulphite-treated tissue DNA was done in a total volume of 15 µL as described in Table S2, PCR assay ID 1 (Region A) and PCR assay ID 2 (Region B). Bands of amplified DNA were ligated with linkers for sequencing on the Roche 454 Titanium FLX system. Amplified products were mixed with amplicons derived from other genes from the same patient and the mixture ligated with bar-coded “MID” linkers (Roche, Cat. 05619211001) so that the sample of origin for each read could be deduced from the sequence. Libraries of pooled amplicons were prepared and sequenced as described previously.^10^ Bisulphite sequencing reads were assigned to individual tissue samples using the bar-code sequences and a custom Python script. Alignment was performed using SHRiMP 1.3.2^30^ and the in silico bisulphite-converted sequence expected from fully methylated DNA. After alignment, the SHRiMP output files were processed using custom R scripts and the fraction of unconverted cytosines at each potential CpG methylation site (identified in Fig. 1) was determined for each sample. A WBC DNA sample (unmethylated control) as well as a 1:1 mixture with fully methylated DNA, were also analyzed for quality control purposes.

### Measurement of *CAHM* methylation

Methylation-specific oligonucleotide primers (Table S2, PCR assay ID 3) were designed to interrogate the methylation status of six CpG sites within the *CAHM* locus (Fig. 1, Region A; Fig. 2A). Methylation-specific qPCR (Roche LightCycler 480 II) was performed on 5 ng bisulphite converted tissue DNA in a total volume of 15 μL as described in Table S2 PCR assay ID 3. A product with a melting temperature peak of 78.4 ± 0.9 °C was characteristic of the methylation-specific *CAHM* amplicon. Standard curves were constructed by assaying a 4-fold serial dilution of positive control CpGenome Universal Methylated DNA, 5000–1.2 pg) in unmethylated control WBC DNA so as to give a total of 5 ng DNA for each sample (Fig. 2B). Standard curves were run in triplicate on each PCR plate to allow Cycle Threshold (Ct) values to be converted to percent methylation (i.e., proportion of fully methylated DNA present). For plasma DNA assays, the input level of DNA, corresponding to 555 µL plasma per PCR assay, is variable. Therefore the standard curve was used to read off the amount (pg) of fully methylated genomic DNA required as input to obtain the Ct value actually measured in the plasma qMSP; the resulting value is expressed as “methylated CAHM, equivalents in genomic DNA,” abbreviated as meCAHMeqgDNA.

### RNA extraction and cDNA preparation

Tissue samples included 10 matched normal/CRC sample pairs purchased from Proteogenex and 7 matched pairs and 5 unmatched specimens from the Adelaide cohort. Tissue and cell line RNA was extracted using Trizol (Invitrogen, Cat. 10296010), and cDNA was prepared from RNA using the QuantiTect Reverse Transcription Kit (Qiagen, Cat. 205311). Since the *CAHM* gene lacks introns, its DNA can therefore serve directly as a template in the qPCR amplification (next section). Each cDNA synthesis was accompanied by a negative control reaction lacking reverse transcriptase to verify the success of the QuantiTect DNA removal step.

### Measurement of CAHM RNA expression

Although we describe our DNA work with reference to the plus strand of the chromosome (e.g., sequences in Figs. 1 and 2A), our descriptions of CAHM expression relate to the RNA (or cDNA second strand) and thus refer to the sequence of the chromosomal minus strand. Reverse Transcriptase quantitative PCR (RT-qPCR) primers (Table S2, PCR assay ID 6) were designed to amplify a 73 bp segment from the 3′-half of the CAHM transcript (Fig. 1A). qPCR was performed on ~6.5 ng cDNA in a total volume of 10 μL as described in Table S2. Assays were performed in triplicate in 384-well plates; *HPRT1* expression (Table S2, PCR assay ID 7) was used as the housekeeper reference for tissue samples, whereas *ACTB* (β-actin) expression (Table S2, PCR assay ID 8) was used for cell lines (except for tissue/cell line cross-comparisons, where *HPRT1* was used for both). A product with a melting peak of 85.5 °C ± 0.6 °C was characteristic of the CAHM RT-qPCR amplicon; absence of amplification was assigned the value of Ct = 52. The amplification efficiency was established using a 2-fold serial dilution of DNA from HT29 cells (9636–1204 gene copies). DNA samples that gave an abnormal qPCR curve or melt profile, or that were found to have DNA contamination (previous section), were eliminated prior to graphing and statistical analysis.

### Statistical analysis

Statistical comparisons for expression of CAHM RNA in matched normal/CRC tissue samples used the paired two-tailed *t* test, as recommended by Instat v3.01 (GraphPad Software Inc.). For analysis of *CAHM* methylation in plasma, *t* test (Mann-Whitney) calculations were performed using GraphPad Prism v5.0d for Mac OS X (GraphPad Software). For density plots of amounts of meCAHMeqgDNA in plasma, samples with “no signal” results were omitted. The empirical density of ln(meCAHMeqgDNA) was estimated separately for normal, early stage and late stage CRC samples. Assuming a Gaussian distribution for the meCAHMeqgDNA values, to give the maximum entropy (the worst case) with the observed mean and variance, the densities were again estimated. Using these densities, we calculated the cumulative distribution function F(x) = P(X ≤ x) and we report 1-F(x) for ln(pg meCAHMeqgDNA) values of 1, 5, and 10. We also report the likelihood ratio relative to the pre-malignant case.

## Supplementary Material

Additional material

## Abbreviations:

CRC: colorectal cancer
meCAHMeqgDNA: methylated CAHM equivalents of genomic DNA
qMSP: quantitative methylation-specific PCR
TSS: transcription start site
WBC: white blood cell

## Notes

10.4161/epi.29046
