# Supplementary material for: CAHM, a long non-coding RNA gene hypermethylated in colorectal neoplasia
Source: Epigenetics. 2014 May 6;9(8):1071–82. doi: 10.4161/epi.29046 (PMC4164492; doi:10.4161/epi.29046)
Supplement: Additional material [file epi-9-1071-s01.pdf]

Landes Bioscience
www.landesbioscience.com

Supplemental Material to:

Susanne K Pedersen, Susan M Mitchell, Lloyd D Graham, Aidan McEvoy, Melissa L Thomas, Rohan T Baker, Jason P Ross, Zheng-Zhou Xu, Thu Ho, Lawrence C LaPointe, Graeme P Young, and Peter L Molloy

CAHM, a long non-coding RNA gene hypermethylated in colorectal neoplasia

Epigenetics 2014; 9(8)
http://dx.doi.org/10.4161/epi.29046

http://www.landesbioscience.com/journals/epigenetics/article/29046/

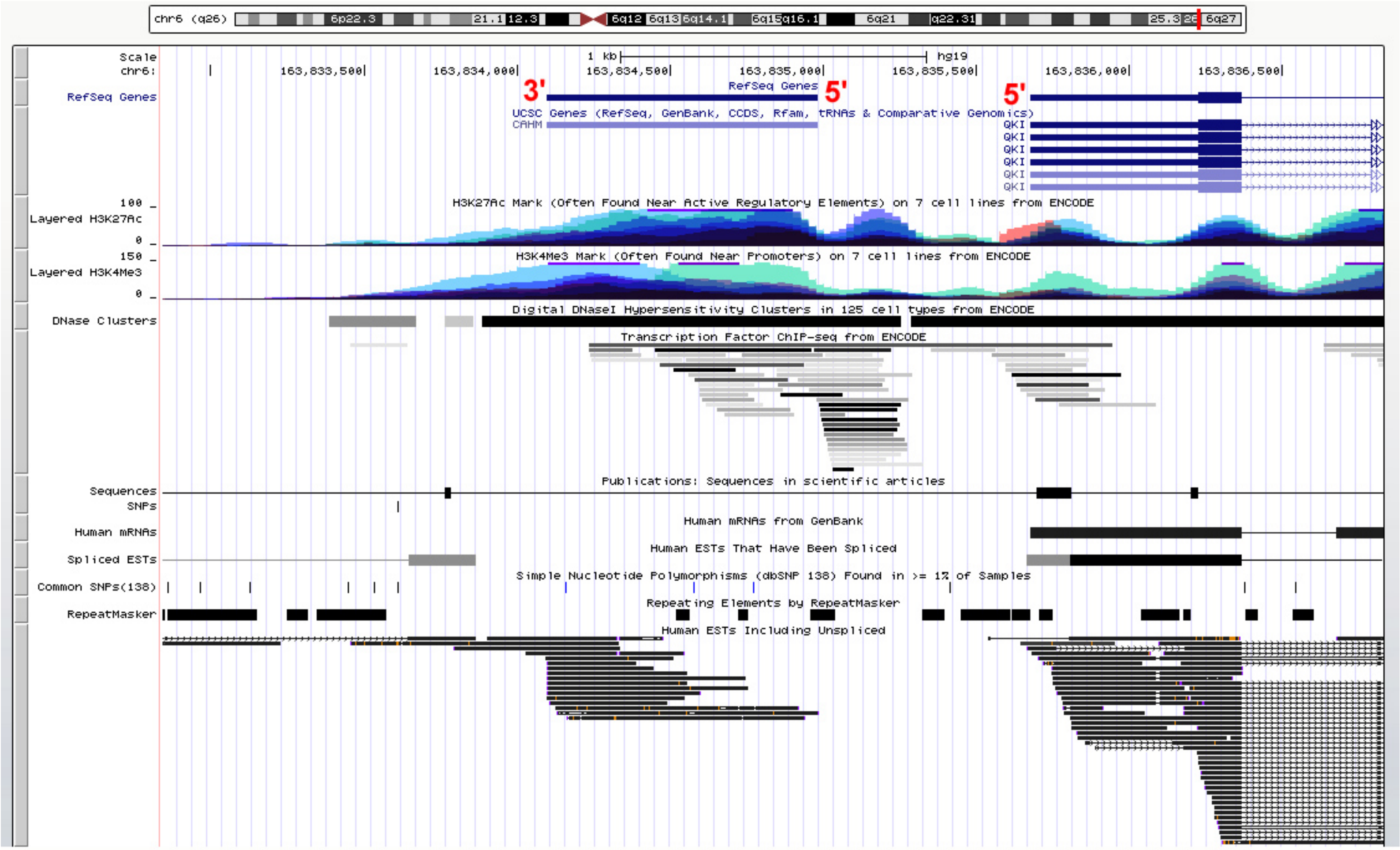

Supp. Fig. S1. The *CAHM* locus (center) and the adjacent portion of *QKI* (right) viewed in the USCC Genome Browser (hg19). The *CAHM* gene is

on the minus strand and therefore the locus appears 3’ to 5’, whereas *QKI* is on the plus strand and runs 5’ to 3’ (bold red annotations). The RefSeq locus for *CAHM* comprises a single exon; consistent with this, almost all of the CAHM ESTs are unspliced (the single exception in the top row of the bottom track may well be an artefact). ENCODE data for the histone modification landscape is shown for H3K27Ac (distinctive of transcribed regions) and H3K4me3 (a classic promoter mark). Both marks seem to span the body of the gene and some distance beyond its official 3’ terminus. It is possible that the core promoter region is under-represnted in ChIP-seq data due to its very high GC content. The ENCODE ChIP-Seq track shows that transcription factor binding is concentrated around and upstream of the 5’ end of the gene, as one would expect. Further detail of the transcription start site(s) and promoter region is not available as 5’-RACE, deletion mapping, etc. have not yet been undertaken.

Supplementary Table S1. Clinical details for 220 blood plasma specimens and measurements of their methylated *CAHM* content

	**Patients,** n (%)	♀/♂	**Mean age** Years (SD)	*CAHM Positive (>3pg/mL threshold)* #, (%)	**Upper & Lower 95% CI of mean** Mean (LCI, UCI)
**Normal**	**74**	**30/44**	**52 (8.6)**	**5 (7)**	**0.8 (0.2, 1.8)**
**Adenoma**	**73**	**33/40**	**55 (5.2)**	**3 (4)**	**9.1 (-8.4, 26.6)**
Lesion <10mm	69	32/37	68 (5.1)	3 (4)	9.6 (-8.9, 28.2)
Lesion ≥10mm	4	1/3	61 (4.7)	0 (-)	0.2 (-0.3, 0.6)
<3 lesions	68	30/38	55 (4.6)	3 (4)	9.7 (-9.0, 28.6)
≥3 lesions	5	3/2	57 (4.5)	0 (-)	-
*TA**	25	12/13	54 (4.2)	1 (4)	25.8 (-27.8, 78.7)
*TVA**	6	3/3	54 (2.0)	1 (17)	0.6 (-1.0, 2.2)
*VA**	1	-/1	63 (-)	0 (-)	-
other	41	18/23	55 (6.2)	1 (2)	0.4 (0, 0.9)
Cancer	**73**	**42/31**	**59 (11.7)**	**40 (55)**	**1893 (175.4, 3611)**
Stage I	12	5/7	64 (11)	5 (42)	21.1 (-14.6, 56.8)
Stage II	21	9/12	57 (13)	11 (52)	33.5 (1.7, 65.2)
Stage III	23	16/7	59 (13)	12 (52)	1438 (-271, 3149)
Stage IV	12	9/3	56 (11)	9 (75)	7886 (-2351, 18124)
Stage Unkn	5	3/2	58 (17)	3 (60)	1523 (-2672 ; 5718)

* *TA* , tubular adenoma; *TVA* tubulovillous adenoma; *VA*, villous adenoma

Supplementary Table S2. PCR assay conditions

PCR ID	Input Material	Gene target	Oligonucleotides	PCR mixture, reaction volume<br>Final concentration	PCR Running conditions<br>Roche' Light cycler 480 model II
Deep Sequencing					
1	BisDNA	*CAHM*	Region A<br>A1: 5'-ATt TGT AAA AAT GTT GAt TTt TGt TTT TtA GAt-3'<br>A2: 5'-TCT TaT TaC ACC TTC CCR TTA TTC Ta-3'	15 μL PCR reaction:<br>0.5U Platinum Taq (Invitrogen, Carlsbad, CA),<br>1X Platinum buffer, 3 mM MgCl2, 200 μM dNTPs,<br>200nM of each primers, 1/100,00-dilution of stock SYBR Green (Invitrogen)	95°C for 2 min,<br>50 cycles of<br>[95°C, 15 s; 56°C, 30 s; 72ºC, 30 s]<br>40°C, 10 s.
2			Region B<br>B1: 5'-GtY GTG tTG tTT Ttt AGt tTt TtA GtA AAT t-3'<br>B2: 5'-CAC RaT aCR aAa aaC Taa Taa aCT TTC CTT a-3'		
Bisulphite conversion dependent quantitative PCR					
3	BisDNA	*CAHM*	(within Region A)<br>1: 5'-GAA GGA AGt ATT TCG AGt ACG AtT GAC-3'<br>2: 5'-CCC GAa CGC AaC GaC TTA a-3'	15 μL PCR reaction:<br>0.5U Platinum Taq, 1X Platinum buffer, 4 mM $MgCl_2$, 200 μM dNTPs, 200nM of each primers, 1/100,00-dilution of stock SYBR Green (Invitrogen)	95°C for 2 min,<br>3 cycles of<br>[95°C, 15 s; 62°C, 15 s; 72ºC, 20 s]<br>50 cycles of<br>[82°C, 15 s; 63°C, 15 s; 72ºC for 20 s]<br>40°C, 10 sec.
4		*ACTB*	1: 5'-GTG ATG GAG GAG GTtT AG tAA GTt-3'<br>2: 5'-CCA ATa aaA CCT aCT CCT CCC TTa A-3'<br>Probe: FAM-5'-ACC ACC ACC CAaCAC ACA aTa aCA AaAC aCa-3'-BHQ1	15 μL PCR reaction:<br>0.75U Platinum Taq, 1X Platinum buffer, 2 mM $MgCl_2$, 200 μM dNTPs, 900nM of each primers, 100nM Probe	95°C for 2 min,<br>60 cycles of<br>[95°C, 10 s; 57°C, 40 s; 72ºC, 10 s]<br>40°C, 10 s.
5		CFF1	1: 5'-TAA GAG TAA TAA TGG ATG GAT GAT G-3'<br>2: 5'-CCT CCC ATC TCC CTT CC-3'	15 μL PCR reaction:<br>0.75U Platinum Taq, 1X Platinum buffer, 3 mM $MgCl_2$, 200 μM dNTPs, 630 nM of each primers,<br><br>1/100,00-dilution of stock SYBR Green<br>OR<br>200nM Probe	95°C for 2 min,<br>50 cycles of<br>[95°C, 10 s; 60°C, 50 s]<br>40°C, 10 s.
RNA expression					
6	cDNA	CAHM	2.1: 5'-AGG GGA GCG TCA GTC GTG CT-3'<br>2.2: 5'-TGC GGC TTC ATT CCC TCA CGG-3'	10 μL PCR reaction:<br>1x GoTaq HotStart Colorless Master Mix (Promega) with SYBR Green I at 1/120,000-dilution of stock and primers (Supp. Table S2) at 600 nM each.	95°C for 2 min,<br>50 cycles of<br>[95°C, 15 sec; 60°C, 60 s]<br>40°C, 10 s].
7		HPRT1	1: 5'-ATG GTC AAG GTC GCA AGC TT-3'<br>2: 5'-GTC AAG GGC ATA TCC TAC AAC AAA CT-3'		
8		ACTB	1: 5'-AGA AGG ATT CCT ATG TGG GGG-3'<br>2: 5'-CAT GTC GTC CCA GTT GGT GAC-3'		

For primers used on bisulphite-treated DNA thymines arising from cytosine conversion are show as lower case “t’ and similarly, complementary adenines as lower case “a”.

Supplementary Table S3. The proportion of methylated *SEPT9* and *CAHM* in DNA from individual neoplastic samples of colorectal tissue

Adenoma		Stage I		Stage II		Stage III		Stage IV	
SEPTIN9	CAHM	SEPTIN9	CAHM	SEPTIN9	CAHM	SEPTIN9	CAHM	SEPTIN9	CAHM
0.44	0.30	55.78	0.02	32.38	27.37	79.57	12.47	73.47	60.19
0.08	**31.92**	4.13	**15.81**	30.12	45.05	78.42	13.27	10.15	4.36
0.83	1.34	9.65	0.02	51.88	5.04	39.38	10.84	5.17	7.25
82.40	67.62	4.62	2.77	71.89	67.47	22.59	10.67	13.31	**28.79**
177.31	111.91	65.97	52.19	34.07	**92.35**	2.17	1.99	29.05	39.06
105.69	34.93	97.62	88.10	45.53	29.04	12.99	5.53	35.58	33.06
37.93	**140.77**	16.57	25.83	4.68	1.93	29.47	16.52	1.60	2.16
125.52	55.32	41.46	0.08	29.26	28.79	33.34	23.22		
3.10	2.24	182.03	0.05	36.99	29.69	79.18	9.46		
61.95	76.27	445.06	0.05	23.31	23.27	40.64	48.01		
389.27	**1168.60**	16.59	19.64	21.75	24.41	21.37	20.22		
		14.89	0.05	123.04	0.05	15.16	1.91		
		16.81	0.00	6.69	7.35	10.01	0.01		
		31.80	17.81	67.25	1.18	9.15	13.06		
		304.29	0.00	70.11	58.51	60.12	35.39		
		80.14	32.79	60.42	0.22	43.70	6.79		
				11.28	0.39	37.53	21.17		
				53.21	67.59	34.40	0.06		
						2.66	4.78		
						6.29	0.95		
						29.86	36.49		
						100.43	88.04		
						n/a	104.09		
						61.97	59.20		
						83.20	35.16		
						53.20	59.44		
						28.43	34.63		
						204.92	109.68		

Table S3 shows the proportion of methylated *CAHM* and *SEPT9* target sequences in individual cancers, measured relative to standard curves derived from methylated and unmethylated diploid DNA, as described in Materials and Methods. *SEPT9* methylation was assayed using the method of de Vos et al. (Clin Chem. 55: 1337-46, 2009).Grey shaded boxes indicate those samples for which the measured proportion of methylated *CAHM* was at least 2 fold higher than the corresponding proportion of *SEPT9*. For the last adenoma sample, consistently high values of methylation have been seen for multiple markers, consistent with deletion of the region covering the PCR reference template (CFF) used to quantify input.
